# A localized vertebral infection model of pyogenic spondylitis induced by *Staphylococcus aureus* in rats

**DOI:** 10.3389/fmed.2025.1643905

**Published:** 2025-09-18

**Authors:** Qinpeng Xu, Guihe Yang, Jiaju Ma, Xingzhi Jing, Fei Jia, Xingang Cui, Jianlong Li, Xiaoyang Liu, Hongdong Tan

**Affiliations:** ^1^Department of Spine Surgery, Shandong Provincial Hospital Affiliated to Shandong First Medical University, Jinan, China; ^2^Department of Spine Surgery, Beijing Jishuitan Hospital, Capital Medical University, Beijing, China; ^3^Shandong Public Health Clinical Center, Shandong University, Jinan, Shandong, China

**Keywords:** pyogenic spondylitis, spinal infection, animal model, *Staphylococcus aureus*, rat

## Abstract

**Background and purpose:**

Pyogenic spondylitis (PS) is clinically challenging and induces disastrous consequences for patients. The pathogenesis of PS is difficult to explore due to a lack of ideal animal models. Thus, we aimed to reproduce the local pathogenesis of PS in an innovative animal model induced by *Staphylococcus aureus*.

**Methods:**

Rats were injected with planktonic *Staphylococcus aureus* (ATCC 25923) and grouped according to different concentrations. We identified the optimal bacterial inoculum concentration based on general physical signs and radiological, hematological, and histological parameters in rats. Models with the optimal bacterial concentration were used to investigate changes in physical, radiological, and inflammatory parameters at different time points.

**Results:**

Our results revealed that infected rats experienced rapid weight loss, high fever, and significantly increased white blood cell count, interleukin 1β, and C-reactive protein (CRP) levels in the short term. Radiographic examination revealed bone damage in groups that received 2 × 10^3^/20 μl, 2 × 10^5^/20 μl and 2 × 10^7^/20 μl bacterial concentrations. The optimal concentration was identified as 2 × 10^5^/20 μl, based on the high survival rate, obvious bone destruction, and inflammation. Histological staining confirmed the living bacteria, inflammatory cells, bone destruction, and scarce bone formation in infected vertebrae.

**Conclusions:**

This study provides an innovative PS animal model that simulates a local iatrogenic vertebral infection and develops innovative and effective strategies for its treatment, but does not simulate the hematogenous dissemination characteristics of most clinical cases of pyogenic spondylitis.

## 1 Introduction

Spinal infections, caused by specific pathogenic microorganisms, usually involve the vertebrae, intervertebral discs, and adjacent perivertebral soft tissue. These infections were classified based on the microbial etiology into pyogenic spondylitis (PS), tuberculous spondylitis (TS), fungal spondylitis, or others (1). PS is the most common infection, whose incidence has been increasing in recent years due to social aging and the misuse of antibiotics (2). PS can lead to sepsis, spinal instability, and neurological deficits, and is becoming a severe and potentially life-threatening disease with a mortality rate of 4 to 29% (3, 4). The lumbar spine is susceptible to PS involvement (58%) (5). The most common pathogen was *Staphylococcus aureus* (*S. aureus*) (6). In attempting to study its pathology, clinical manifestations, and treatment strategies, we have only partially recognized this disease. Therefore, it is pivotal to comprehensively explore the pathogenesis of PS and its influence on patients. However, the lack of a suitable animal model critically hinders further exploration of PS prevention and treatment (7).

In the pathogenesis of PS, bacteria from a distant infectious lesion may survive in the terminal microvasculature beneath the vertebral endplate (8). The rapid growth of local bacteria leads to vessel occlusion, ischemic necrosis, osteonecrosis, and vertebral osteomyelitis. Local bacteria proliferate and damage vertebrae, cartilage endplates, and intervertebral discs owing to invasive enzymes (such as hyaluronidase secreted by *S. aureus*), resulting in pyogenic discitis (9). However, there is no suitable animal model to simulate PS originating from the primary vertebral infection.

Animal studies of osteomyelitis have been reported, including the traumatic osteomyelitis model in rats (10) and the periprosthetic joint infection model in rabbits (11). The imaging and pathological changes were narrated in these studies. Unfortunately, these models cannot appropriately simulate the characteristics of spinal infection. Bierry and Chen created models of PS in rabbits and in canines, respectively (12, 13). In these two studies, animals were injected with a bacterial suspension into the intervertebral space, which differs from the pathogenesis of human PS. Sweet created a rabbit infectious model by laminectomy, which can be used to mimic surgical site infection after metal implantation (14). These models have obvious limitations, including high technical requirements and poor imitation. PS imposes a great burden on families and society, thus innovative animal models are necessary to further explore the pathogenesis of PS and potential treatment strategies.

Rats are commonly used due to their low cost, minimal individual differences, antibiotic tolerance, and manageable body size (15). It is vital to establish an animal model to reproduce the pathogenesis of PS in order to investigate the mechanisms underlying inflammation and bone metabolism (16). The objective of this study was to establish a new, reliable animal model to precisely mimic the pathogenesis of PS, and further explore novel anti-infective strategies.

## 2 Materials and methods

### 2.1 Animal selection and grouping

This study was approved by the Animal Ethics Committee and Institutional Review Board at our hospital (NO. SDNSFC 2023-0198). Seventy-five male Wistar rats aged 8 weeks old were used in this study. The mean weight of the animals was 250 g. The animals were housed in individual cages at least 6 days before surgery and had free access to food and water. To determine the optimal *S. aureus* concentration, rats were randomly divided into five groups. The experimental groups were administered a 20 μl *S. aureus* suspension of varying concentrations: 1 × 10^3^ (G1 group, *n* = 7), 1 × 10^5^ (G2 group, *n* = 7), 1 × 10^7^ (G3 group, *n* = 7), 1 × 10^9^ colony forming units (CFU)/ml (G4 group, *n* = 7). The control group received 20 μl phosphate buffer saline (PBS). Postoperatively, body weight and anal temperature was measured daily. Rats reached the endpoint of humanitarian execution when weight loss was >20% or anal temperature was < 36 °C.

### 2.2 Preparation of bacterial inoculum

*Staphylococcus aureus* (ATCC 25923) was incubated in trypticase soy agar (TSA) medium at 37 °C for 18 h. After 18 h, *S. aureus* was harvested and resuspended in sterile PBS at a concentration of 1 × 10^9^ CFU/ml according to optical density measurements. The suspension was diluted into various bacterial concentrations (1 × 10^3^, 1 × 10^5^, 1 × 10^7^ CFU/ml). Referring to physical signs, bone destruction, inflammatory markers and survival rates of animals after injection, a preliminary experiment was first performed to identify the appropriate concentration of bacterial inoculum.

### 2.3 Surgical procedure

**Figure 1:**
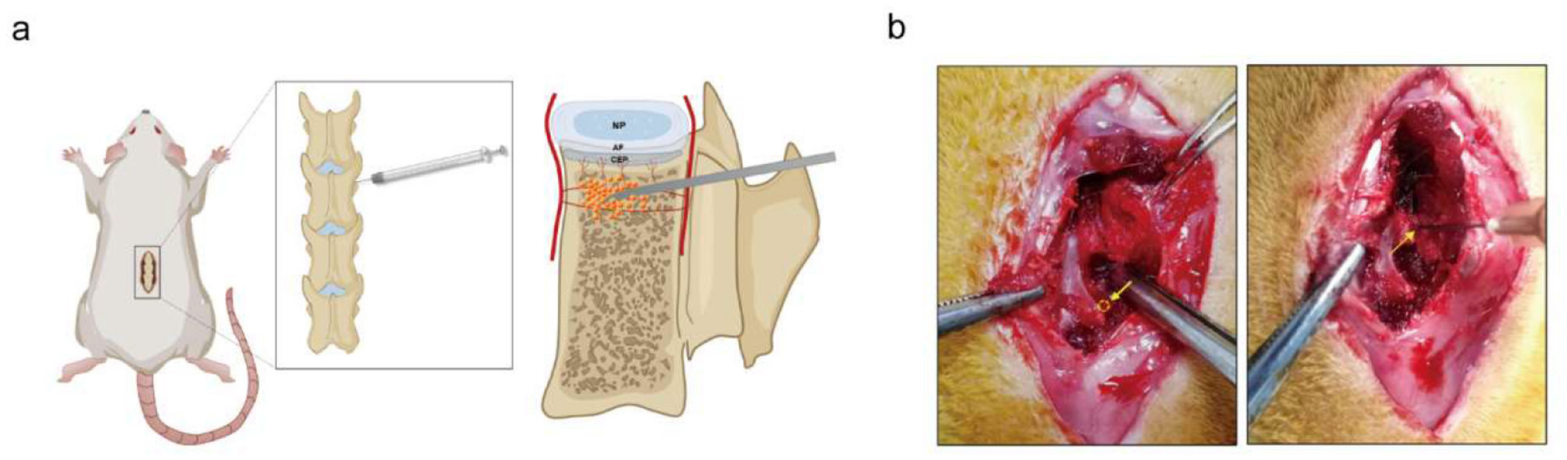
Surgical procedure diagram. **(a)** The diagram indicates the surgical procedures conducted during the study. The lumbar spine of the rat was exposed. The puncture point was the junction of the transverse and articular processes and the channel reached the anterior part of the vertebra. The bacterial solution was injected into the vertebral body (below the endplate) using a 25 μl syringe. **(b)** Operative feature. Yellow arrows show the insertion position.

The anal temperature and weight of each rat were measured before surgery. To prepare a 100% Avertin stock solution, 10 g of tribromoethanol was dissolved in 10 mL of tertiary amyl alcohol in a centrifuge tube by gently shaking by hand until fully dissolved. The solution was then filtered through a 0.22 μm membrane. The 100% Avertin stock was diluted with 0.9% NaCl physiological saline to a final concentration of 2.5% (1:40 dilution). Avertin (2.5%) was administered via intraperitoneal injection at a dose of 250 mg/kg for each rat. After shaving and sterilization with povidone-iodine, the lumbar vertebra was exposed posteriorly. The skin and fascia were incised sequentially with tissue scissors and a scalpel. Thereafter, the right lamina, facets, and transverse processes were exposed. The puncture point was located at the transition point between the superior articular process and the transverse process. First, the outer layer of the puncture point was opened with a needle (0.45 mm × 16 mm) from a 1 ml syringe, then the puncture channel was made by a needle (0.33 × 12 mm) through the vertebral pedicle. The channel with a depth of 3 mm ended at the anterior section under the endplate. Subsequently, a 20 μl bacterial solution was injected into the vertebral body through the established channel. After injection, the puncture site was sealed using bone wax. Finally, the muscle, fascia, and skin were closed layer by layer after washing (Figure 1). All procedures were conducted aseptically. No antibiotic was administered preoperatively, intraoperatively, or postoperatively.

### 2.4 Blood assays

Blood was collected by cardiac sampling at the endpoints of 7, 14, 21, and 28 days postoperatively. WBC number and morphological differences were determined from blood smears using CellaVision^®^ DM9600. Serum was obtained from blood by centrifugation at 2000 × g for 15 min. According to the manufacturer's instructions, the CRP and interleukin 1β (IL-1β) levels were determined using an enzyme-linked immunosorbent assay (ELISA) (Abcam, Cambridge, Massachusetts, USA).

### 2.5 Radiographic evaluation

Rats were executed at their endpoints post operation. The lumbar spine was harvested and fixed in 4% paraformaldehyde, then scanned using microcomputed tomography (μCT) (Scanco Viva-CT80, Scanco Medical AG, Basserdorf, Switzerland). The data processing and 3D reconstruction were performed using Built-in software. Vertebral destruction was calculated using embedded 3D measurement techniques. Spinal destruction was scored using a modified An and Friedman scoring system (Table 1) by three individuals blinded to the experimental protocol.

**Table 1:** Modified An and Friedman scoring system.

**Score**	**Osteolysis**	**Osteogenesis**	**Vertebral body wall destruction**	**Paravertebral abscess**
0	Not present	Not present	Not present	Not present
1	Unifocal lesion	Focal osteosclerosis within the vertebra	Single focal lesion	Focal abscess involved in a single vertebra
2	Multifocal lesions	Generalized osteosclerosis within the vertebra	Multiple focal lesions	Generalized abscess involved in a single vertebra and adjacent disc
3	Lesions involved in two vertebrae	Osteogenesis evident outside the vertebral body	Lesions exceeding half of the vertebra	Abscess involved in two vertebrae
4	Lesions involved in multiple vertebrae (>2 vertebrae)	Significant osteogenesis involved in at least two vertebrae	Extensive lesions involved in at least two vertebrae	Abscess involved in multiple vertebrae (>2 vertebrae)

### 2.6 Histological analysis

After μCT measurement and decalcification, the infected vertebrae and adjacent discs were separated and embedded in paraffin. Next, samples were sectioned axially at 5 μm thickness to obtain anatomical sections, which were deparaffinized, rehydrated, and stained with hematoxylin and eosin (H&E) according to routine protocols. Goldner trichrome staining was used to assess bone mineralization. In addition, Gram staining was performed to determine bacterial infection in the tissue. Immunohistochemical staining was used to evaluate the expression of inflammatory factors. Sections were blocked with 5% BSA at 37 °C for 30 min.

### 2.7 Statistical analysis

The data is presented as mean ± standard deviation (SD). The Student's t-test was used to evaluate differences between the two groups. One-way analysis of variance (ANOVA) was used when there were more than two groups (SPSS V19.0, SPSS Inc., Chicago, IL, USA). *P*-values < 0.05 were deemed significant.

### 2.8 Euthanasia method

Five to eight rats were placed in the euthanasia box. The CO_2_ cylinder switch was turned on, and CO_2_ was introduced into the box at a rate of 10%−30% of the box volume per minute, until the animals no longer moved, breathes, or exhibited pupil constriction. The CO_2_ cylinder switch was then turned off, and the animals were observed for an additional 2–3 minutes to confirm death.

## 3 Results

### 3.1 Vital signs and mortality

**Figure 2:**
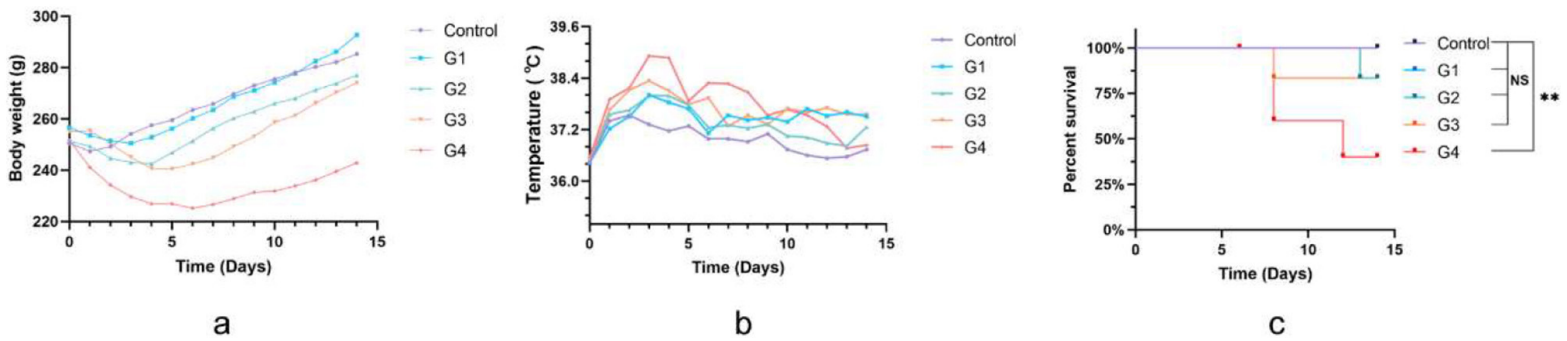
Physical markers of pyogenic infections. **(a, b)** Body weight and temperature of rats among different groups after surgery. **(c)** Survival rate curves of rats after surgery in different groups. Data are presented as mean ± standard deviation (SD). ns-no significance, **P < 0.01.

All rats were observed for 14 days after surgery. There was no statistically significant difference in body weight between the control group and the G1–G3 groups after surgery. However, rats in the G4 group, receiving the highest dose of bacteria (10^9^ CFU/ml), experienced the greatest weight loss after bacterial inoculation. All groups showed high body temperature on the first day post-surgery, but the mean temperature of the G1–G4 group peaked 3–5-day post operation and remained significantly higher than that of the control group. However, three rats in the G4 group showed cachexia, rapid weight loss, abnormally decreased temperature, and eventually died. The mortality rate of the G4 group was 42.86%. In contrast, the survival rates of the control group and the G1–G3 groups were greater than 80% (Figures 2a-c).

### 3.2 Blood assays

WBC and CRP are well-established biomarkers for detecting infections. Blood smear analysis revealed a significantly increased WBC in the infected group compared with the control group, while there were no significant differences between infected groups. The CRP level in the G3 and G4 groups was higher than that in the control group. Due to significant individual variations, the CRP level in the G1 and G2 groups showed no statistical significance compared with that of the control group. Additionally, the G3 and G4 groups displayed higher IL-1β levels compared with the control group and G1 and G2 groups, which was similar to observed CRP expression levels (Supplementary Figures S1a-c).

### 3.3 Radiographs

**Figure 3:**
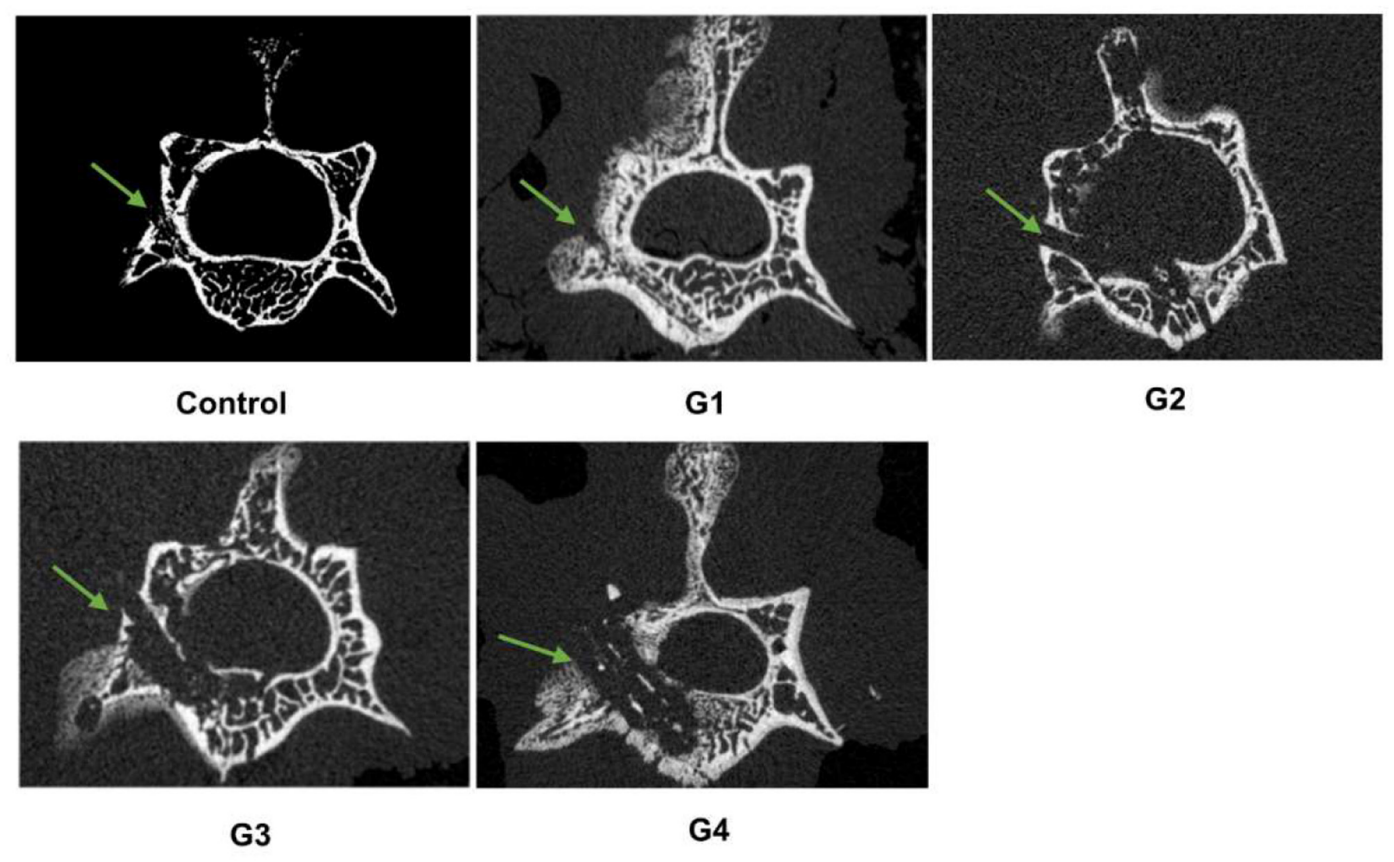
Imaging findings of pyogenic infections. Micro-CT imaging shows bone destruction in different groups two weeks after surgery. Bone infections are indicated by green arrows.

μCT scans showed that G3 and G4 groups exhibited significant bone destruction, hyperostosis, and sclerosis, while G1 and G2 groups had lower levels of bone destruction compared to G3 and G4 groups, and the control group had narrow channels surrounded by obvious sclerotic bone (Figure 3). Based on the modified An and Friedman rating system (Table 1), the scores of G3 and G4 groups were significantly higher than that observed in G1 and G2 groups. The control group displayed the minimum score (Supplementary Figure S2).

### 3.4 Identifying the optimal bacterial concentration

Based on physical signs, bone destruction on CT images, and levels of inflammatory markers, we aimed to identify the optimal bacterial concentration to induce PS. Although obvious bone destruction was observed in both G3 and G4 groups, the maximal concentration in the G4 group was not optimal due to the excessive weight loss and low survival rate. Despite improved survival rates, the G1 and G2 groups showed lower imaging scores and inconsistent levels of inflammatory markers. However, some rats in G1 and G2 groups were not successfully infected by the any bacterial suspension. Therefore, we ultimately identified the 10^7^ CFU/ml (20 μl) in the G3 group as the optimal inoculation concentration for subsequent experiments.

### 3.5 Histology and bacteriologic analyses

**Figure 4:**
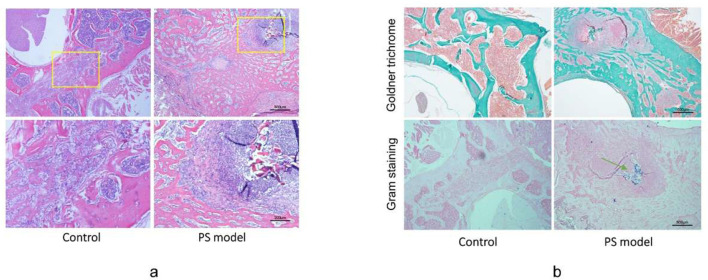
Histological evidence of pyogenic spondylitis. **(a)** Hematoxylin and eosin (H&E) staining of vertebrae in Control and G3 groups revealed obvious bone destruction and massive inflammatory cell infiltration. Scale bar = 500 μm and 200 μm. **(b)** Goldner trichrome staining indicated milder bone-forming in PS rats compared with that in the control group. Gram staining indicated the colonies of injected *Staphylococcus aureus* in the PS model group. The green arrowhead indicates gram-positive bacterial colonies. Scale bar = 500μm.

We performed H&E staining for spinal tissues after rat execution. The control group showed normal trabecular morphology, puncture channels, and surrounding sclerosis (Figure 4a). Rats infected by bacterial suspension showed obvious bone destruction filled with fibrous tissue, massive inflammatory cell infiltration, and periosteal reaction (Figure 4b). According to the histological scoring criteria of bone infection proposed by Smeltzer et al. (17), the scores in the G3 and G4 groups were significantly higher than that observed in other groups (Supplementary Figure S3).

In addition, we performed Goldner staining to evaluate osteogenesis in spinal infection. Mild mineralization was observed in G3 and G4 groups using Goldner staining. In contrast, the active bone-forming response was observed in the control group and in certain rats in G1 and G2 groups. These results were consistent with the μCT imaging findings (Figure 4b). Gram staining performed at execution points revealed the presence of gram-positive bacteria around the surgical puncture site (Figure 4b). The infected vertebrae were collected and inoculated. Numerous colonies of bacteria were observed and identified as *S. aureus* (ATCC25923) using mass spectrometry analysis. The bacterial culture showed no bacteria in the liver and blood from the PS group, which indicated that the animal model displayed local infection without systemic infection.

### 3.6 Dynamic changes to physical characteristics

**Figure 5:**
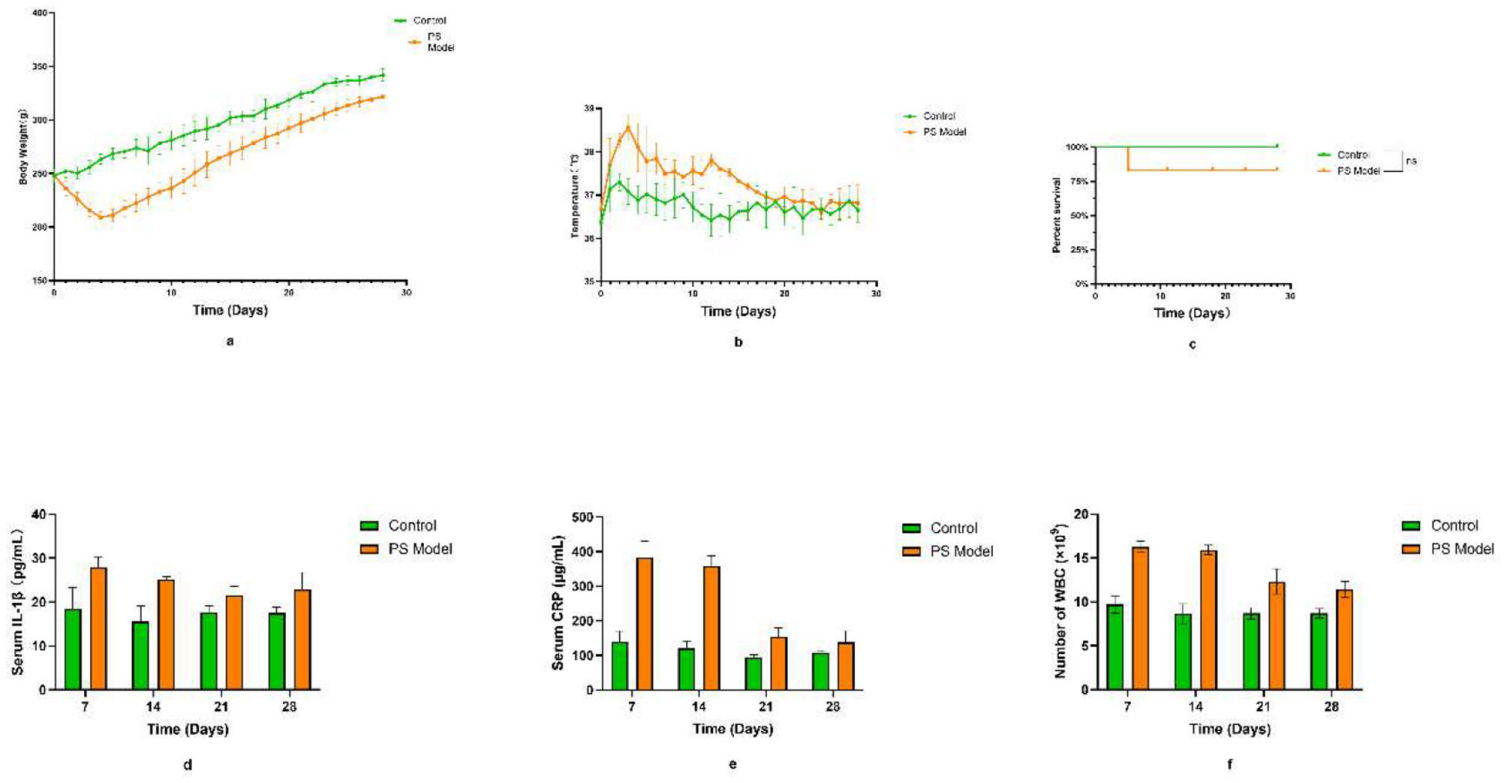
Vital signs of rats within 28 days. **(a)** Body weight and **(b)** temperature of rats among different groups after surgery. **(c)** Survival rate curves of rats after surgery in different groups. ns-no significance. **(d)** Serum interleukin-1β (IL-1β), **(e)** C-reactive protein (CRP), and **(f)** white blood cell (WBC) values in different groups of rats 28 days after surgery.

After confirming the inoculating concentration, we monitored consecutive changes to physical characteristics and inflammatory parameters for 4 weeks. The PS group presented with higher body temperatures than the control group from 3 to 15 days post operation, but there was no statistical difference 20 days after injection. The body weight of the infected rats was lower than that of the control group. In addition, the survival rates at the endpoint were 100% and 80% in the control and PS groups, respectively (Figures 5a-c). Inflammatory markers showed dynamic changes in both groups after surgery. Moreover, CRP levels in the PS group were higher than those observed in the control group (Figure 5e). We observed similar trends in IL-1β levels, WBC count, and CRP levels (Figures 5d, f).

## 4 Discussion

In recent years, the incidence of PS has increased due to the increased life expectancy of patients with chronic diseases and the misuse of antimicrobial drugs (18). PS generally involves bone and intervertebral discs, thereby inducing several complications that may lead to spinal instability, vertebral collapse and kyphosis, and neurological dysfunction. These complications are common and unique in PS. Moreover, discitis and epidural abscess is a specific presentation of PS. However, the vertebra has no medullary cavity but adjacent discs, which are distinct to the tubular bone. PS is characterized by inflammatory infiltration, bone destruction, and intervertebral or intravertebral abscess, leading to inflammatory reactions, spinal instability, and neurological dysfunction (Supplementary Figure S4). Most animal models of osteomyelitis focus on the bones of the limbs, such as the tibia and femur (10, 19). Most of these models were used for implant infection or for the evaluation of antibiotic therapy in the tubular bone. Unfortunately, these findings were not suitable for PS due to the distinct anatomy and pathogenesis.

There are several animal models for spinal infection. Chen et al. (13) reported a canine model of intervertebral disc infection and evaluated it through microbiology, imaging, and histopathology. Owing to its high cost and the difficult surgical procedure, this animal model was not widely used for anti-infection strategies. Rabbits are commonly used as animal models for spinal infections. Guiboux et al. (20). and Bierry et al. (12). produced a PS animal model by injecting *S. aureus* into the intervertebral disc and Bostian et al. established a similar model in rats (21). Small animal models are often preferred due to their lower costs and ease of handling. Ofluoglu et al. (22) established a PS infection model in rats by inserting a pedicle screw with *S. aureus*. However, these models were inconsistent with the pathogenesis of human PS.

In adults, the disc is avascular and resistant to some bacteria, but *S. aureus* and other enzyme-producing bacteria can spread directly to the intervertebral disc by disrupting the endplate. PS is typically initialized by bacterial colonization in slow-flowing capillaries beneath the vertebral endplate and is characterized by localized bone erosion in this region. Thus, infection emanating from the vertebra is similar to the pathogenesis of PS. In this study, the pathogenic bacterium was injected into the vertebra under the endplate, which agrees with the local pathogenesis of PS (8). Other studies have found that injury can significantly increase the likelihood of bone infection (23). In this animal model, we first induced vertebral bone injury by puncture and then inoculated bacteria into the lumbar vertebral body of rats, which is consistent with the pathogenesis of PS. Imaging and histological analyses showed that infected rats exhibited early-stage vertebral destruction, which subsequently progressed and spread to the intervertebral disc through the compromised endplate, closely mimicking the local pathological progression of human PS.

We identified the optimal inoculum concentration (10^7^ CFU/ml) of *S. aureus* based on basic vital signs, imaging, hematology, histology, and bacteriology. Infected rats exhibited high fever and weight loss in the first week, then returned to normal temperature and weight around the second week after inoculation. The levels of WBC and CRP also increased first and then decreased after infection. In addition, μCT images showed significant bone destruction and slight osteogenesis surrounding the lesion. H&E staining showed bone destruction without normal bone trabeculae in the vertebrae, which were replaced by fibrous tissue and inflammatory cells. Meanwhile, Gram staining and bacterial culture results showed the activity of *S. aureus* in the PS model group. In summary, the above results demonstrate that this animal model can reliably mimic the local pathogenesis of PS in humans.

This study has several limitations. Firstly, we conducted CT scanning and inflammatory tests on the 14th day after inoculation when the rats displayed obvious bone destruction. It should be further confirmed whether bacteremia occurs earlier after inoculation. Earlier evaluation may help to understand the pathogenesis during PS onset. Secondly, the PS model in this study was only tested on the most common bacterium. It's necessary to re-evaluate its responses to other pathogens. Meanwhile, this localized vertebral infection model is also not applicable to hematogenous spondylitis because of the different pathogenesis. This model simulates a localized, iatrogenic form of vertebral infection and does not replicate the hematogenous dissemination that characterizes most clinical cases of pyogenic spondylitis.

## 5 Conclusions

In conclusion, we successfully established a PS animal model by injecting *S. aureus* into the vertebral body below the endplate. This is only a local iatrogenic vertebral infection and does not reflect the hematogenous dissemination characteristics of most pyogenic spondylitis, but it helps us better understand its local pathogenesis and find effective treatments.

## Data Availability

The original contributions presented in the study are included in the article/Supplementary material, further inquiries can be directed to the corresponding authors.

## Supplementary material

Supplementary Figure S1 Hematological evidence of pyogenic infections. **(a-c)** White blood cell (WBC), C-reactive protein, and serum interleukin-1β (IL-1β) values in different groups of rats two weeks after surgery. ^**^*P* < 0.01, ^***^*P* < 0.001, ^****^*P* < 0.0001.

Supplementary Figure S2 Imaging scores between different groups. Data are presented as mean ± standard deviation (SD). ns-no significance, ^****^*P* < 0.0001.

Supplementary Figure S3 Histological scores of vertebrae in different groups. ^**^*P* < 0.01, ^****^*P* < 0.0001.

Supplementary Figure S4 **(a, b, c)** Obvious bone destruction on computed tomography images. **(d, e)** Inflammatory infiltration and intervertebral abscess on magnetic resonance imaging.
