# Supplementary figures and images for: A localized vertebral infection model of pyogenic spondylitis induced by Staphylococcus aureus in rats
Source: Front Med (Lausanne). 2025 Sep 18;12:1643905. doi: 10.3389/fmed.2025.1643905 (PMC12488691; doi:10.3389/fmed.2025.1643905)

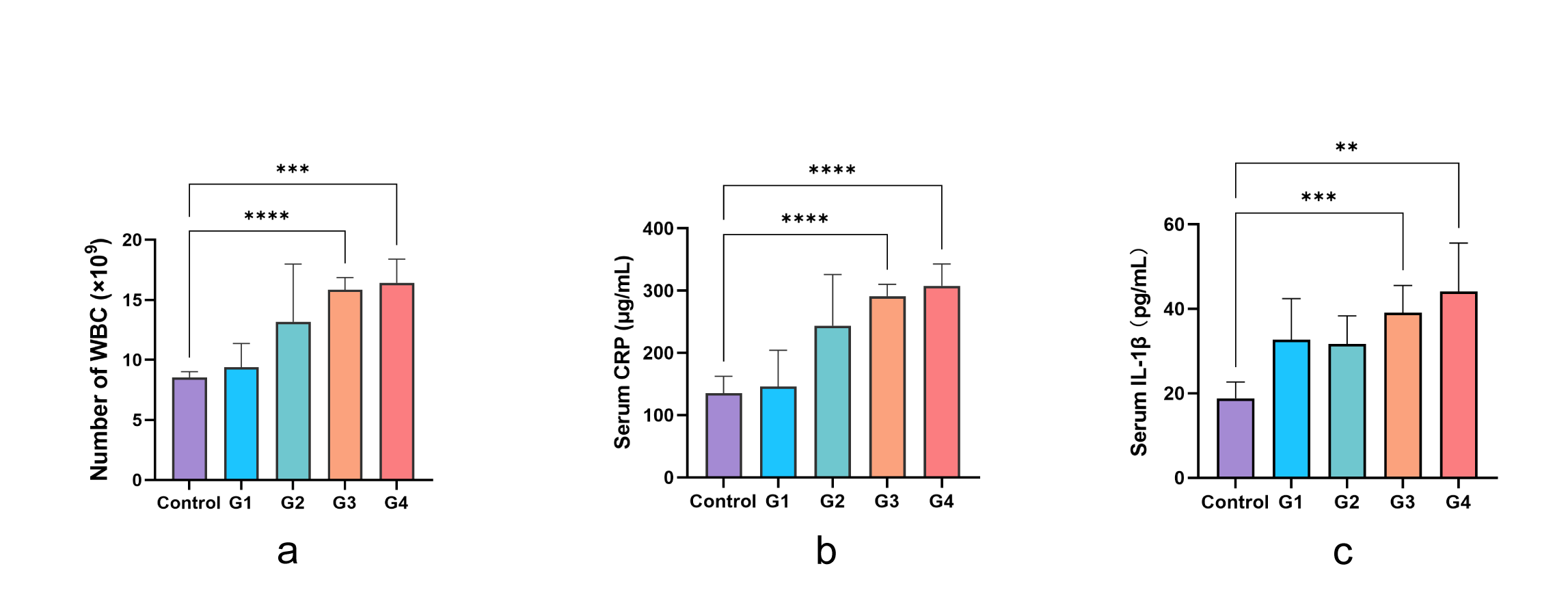

Supplement: Supplementary Figure S1 — Hematological evidence of pyogenic infections. (a-c) White blood cell (WBC), C-reactive protein, and serum interleukin-1β (IL-1β) values in different groups of rats two weeks after surgery. **P < 0.01, ***P < 0.001, ****P < 0.0001. [file Image_1.tif]

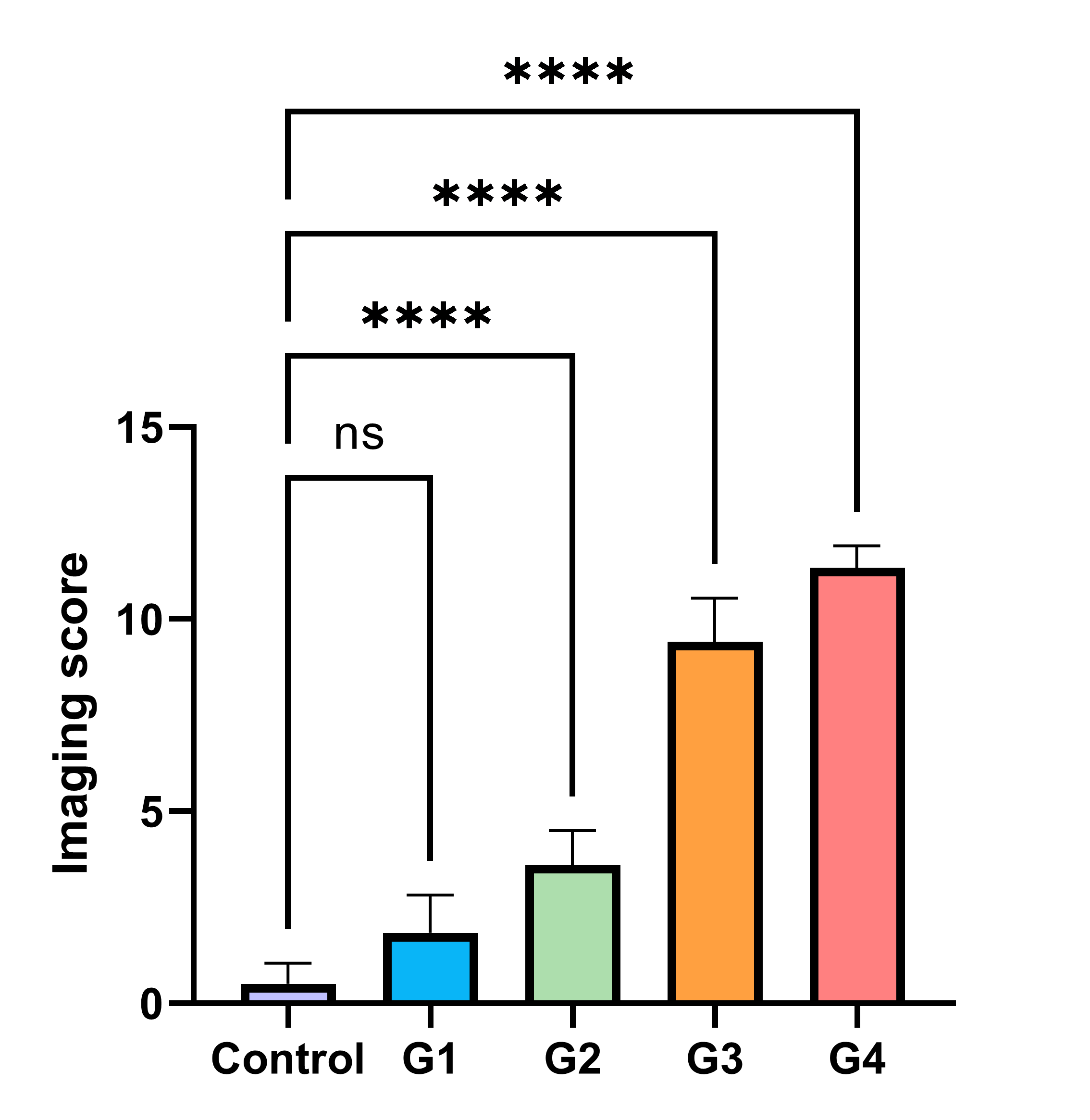

Supplement: Supplementary Figure S2 — Imaging scores between different groups. Data are presented as mean ± standard deviation (SD). ns-no significance, ****P < 0.0001. [file Image_2.tif]

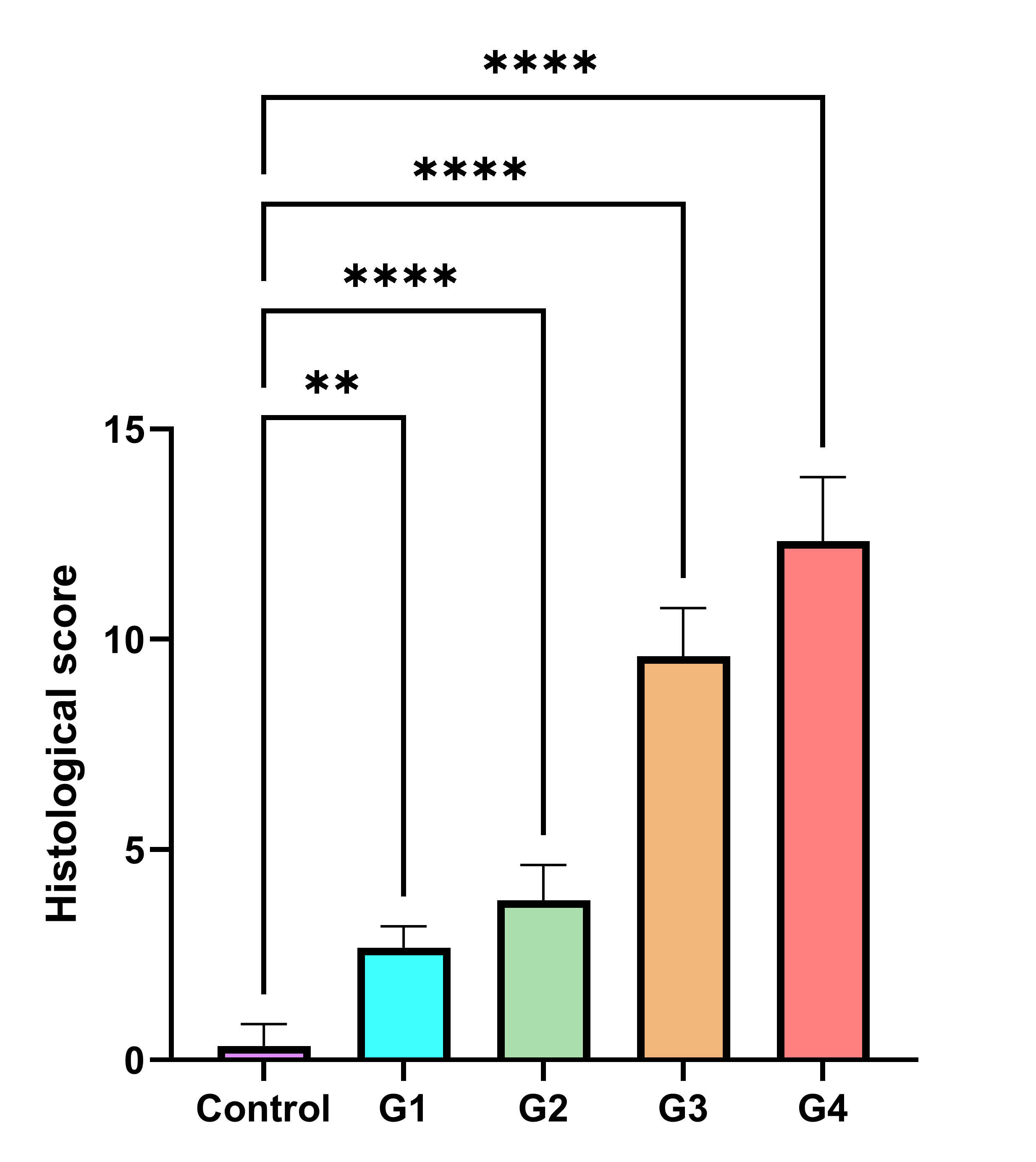

Supplement: Supplementary Figure S3 — Histological scores of vertebrae in different groups. **P < 0.01, ****P < 0.0001. [file Image_3.tif]
